# Genome Variability in Artificial Allopolyploid Hybrids of *Avena sativa* L. and *Avena macrostachya* Balansa ex Coss. et Durieu Based on Marker Sequences of Satellite DNA and the ITS1–5.8S rDNA Region

**DOI:** 10.3390/ijms25105534

**Published:** 2024-05-19

**Authors:** Alexandra V. Amosova, Alexander A. Gnutikov, Alexander V. Rodionov, Igor G. Loskutov, Nikolai N. Nosov, Olga Yu. Yurkevich, Tatiana E. Samatadze, Svyatoslav A. Zoshchuk, Olga V. Muravenko

**Affiliations:** 1 Engelhardt Institute of Molecular Biology of Russian Academy of Sciences, 119991 Moscow, Russia; 2 Komarov Botanical Institute of Russian Academy of Sciences, 197376 St. Petersburg, Russia; 3 Federal Research Center N.I. Vavilov All-Russian Institute of Plant Genetic Resources (VIR), 190000 St. Petersburg, Russia

**Keywords:** *Avena sativa*, *Avena macrostachya*, interspecific hybridization, allopolyploid genome composition, ribotypes

## Abstract

Artificial hybrids between cultivated *Avena* species and wild *Avena macrostachya* that possess genes for resistance to biotic and abiotic stresses can be important for oat breeding. For the first time, a comprehensive study of genomes of artificial fertile hybrids *Avena sativa* × *Avena macrostachya* and their parental species was carried out based on the chromosome FISH mapping of satellite DNA sequences (satDNAs) and also analysis of intragenomic polymorphism in the 18S–ITS1–5.8S rDNA region, using NGS data. Chromosome distribution patterns of marker satDNAs allowed us to identify all chromosomes in the studied karyotypes, determine their subgenomic affiliation, and detect several chromosome rearrangements. Based on the obtained cytogenomic data, we revealed differences between two *A. macrostachya* subgenomes and demonstrated that only one of them was inherited in the studied octoploid hybrids. Ribotype analyses showed that the second major ribotype of *A. macrostachya* was species-specific and was not represented in rDNA pools of the octoploids, which could be related to the allopolyploid origin of this species. Our results indicate that the use of marker satDNAs in cytogenomic studies can provide important data on genomic relationships within *Avena* allopolyploid species and hybrids, and also expand the potential for interspecific crosses for breeding.

## 1. Introduction

The common oat (*Avena sativa* L., 2n = 6x = 42, AACCDD) is one of the most cultivated crops worldwide and a valuable resource both for human consumption and livestock feed [1,2]. This species is characterized by the large and complex allopolyploid genome (1C = 12.8 Gb) that includes about 121 thousand protein-coding genes [3,4]. The history of the origin of the *A. sativa* genome is very complicated. About five or six whole-genome duplications had occurred before the diversification of the BOP clade (subfamilies Bambusoideae, Oryzoideae, and Pooideae) of the family Poaceae took place, and reconstruction of the ancestral grass genome was completed [5,6,7,8].

The genus *Avena* L. is characterized by its complex history of polyploidy, lineage divergence and evolution of chromosomes and genome [9,10,11,12]. Comprehensive studies on molecular genetics, as well as the chromosome structure of cultivated and wild *Avena* species, might provide important information for crop improvement via interspecific hybridization. The species relationships have been intensively studied with the use of both molecular and cytological approaches. Several molecular genetic markers, namely RFLP (Restriction Fragment Length Polymorphism), AFLP (Amplified Fragment Length Polymorphism), SRAP (Sequence-Related Amplified Polymorphism), and SSRs (Single Sequence Repeats), and also the retrotransposon molecular markers IRAP (inter-retrotransposon amplified polymorphism) and REMAP (retrotransposon-microsatellite amplified polymorphism) were used to study genomic diversity within the genus *Avena* [13,14,15,16]. Phylogenetic correlations within *Avena* were evaluated by analyzing the primary nucleotide sequences of the ITS1 and ITS2 (Internal Transcribed Spacers) rDNA regions and also the sequences of FL intron2 (second intron of the nuclear gene FLORICAULA/LEAFY) in species with distinct genome composition [17,18,19,20,21].

The genus *Avena* comprises up to 30 recognized diploids (genomes A or C, 2n = 2x = 14), tetraploids (genomes AB or AC, 2n = 4x = 28), and hexaploids (genome ACD, 2n = 6x = 42) [9,22,23], but evolutionary history and phylogeny of *Avena* still remain under study [11,24]. The C genome is structurally different from other *Avena* genomes [25]. Both B and D genomes are similar to the A genome [26,27], and they are supposed to be derived from the ancestral A genome [28]. Divergence took place among the ancestral diploid *Avena* species, resulting in A, C, and D genome lineages. Then, about 0.5 million years ago, the hybridization between a paternal Al/As-genome diploid ancestor and a maternal CD-genome tetraploid resulted in the formation of the hexaploid ACD genome of common oat [4,8,29]. It was assumed that AB tetraploids arose as a result of some events involving autopolyploidization of A diploid species [11].

Currently, besides *A. sativa*, only three other species are cultivated, namely diploid (genome A) *A. strigosa* Schreb., tetraploid (AB) *A. abyssinica* Hochst., and also hexaploid (ACD) *A. byzantina* K. Koch [16,23]. Among them, *A. sativa* is a valuable temperate crop recommended by nutritionists because its consumption helps reduce blood cholesterol levels and heart disease risks [30,31]. In addition, some wild species of *Avena* possess important agronomic traits, which make them highly potential for oat breeding programs [14]. One these economically interesting species is *A. macrostachya* Balansa ex Coss. et Durieu (2n = 4x = 28), which is a relict wild oat species endemic to the Atlas Mountains [32,33]. It is also the only perennial cross-pollinating *Avena* species which occupies an isolated position within the genus based on the analysis of phenotypic, developmental, and reproductive characters [23]. *A. macrostachya* possesses important resistance genes desirable to be transferred to cultivated species (e.g., *A. sativa*), which include extreme winter hardiness and resistance to some diseases and pests (powdery mildew, crown and stem rust, barley yellow dwarf mosaic virus, soil-borne mosaic virus, and the aphid *Rhopalosiphum padi*) [34,35,36].

To create novel material for oat introgression breeding and develop new winter-hardy oat varieties resistant to biotic stresses, a series of experiments on crossing *A. macrostachya* with various *Avena* species was conducted. Particularly, it was shown that *A. sativa* × *A. macrostachya* octoploids and decaploids were effective sources of winter hardiness for hexaploid oats [36,37,38,39]. The analyses of chromosome pairing together with the results of crossings revealed the high degree of allosyndesis in which C-genome-bearing species were combined with *A. macrostachya*, and it was assumed that the C genome was closer to *A. macrostachya* than the A genome [40]. The close relationship of *A. macrostachya* with a C-genome-carrying group of *Avena* was later confirmed by studying meiosis in triploid hybrids of *A. macrostachya* with *A. damascena* Rajhathy et B.R. Baum and *A. ventricosa* Balansa [41]; analyzing the DNA sequence of the extracellular domain of *A. sativa* receptor-like kinase (ALrk10) gene [42]; and comparatively studying ITS1–5.8S–ITS2 [17,18] and 5S rRNA gene sequences [43]. This close relationship makes it difficult to discriminate chromosomes in karyotypes of hybrids between *A. macrostachya* and *Avena* species bearing the Cgenome by genomic in situ hybridization (GISH), which is a widely used technique to detect alien chromosomes in plant hybrid genomes [44]. It was reported, however, that the genome of *A. macrostachya* differed from the C genomes of diploid *Avena* species in chromosome morphology and distribution of heterochromatin [32,45,46], and a special symbol, CmCm, was later assigned to the genome of *A. macrostachya* [17].

The investigation of the oat genome structure involved the analysis of meiotic chromosome pairing, patterns of C-banding, genomic in situ hybridization (GISH), and fluorescence in situ hybridization (FISH) with the use of various DNA probes, e.g., 35S and 5S rDNA, probes specific to oat A (pAs120a) and C (pAm1) genomes, and also microsatellite motifs [9,47,48,49,50]. Moreover, chromosome and genome diversity within *Avena* were studied with the use of NGS technologies, including chromosome-scale assemblies, and also FISH mapping of different families of satellite sequences (satDNAs) identified in whole-genome sequence reads [51,52,53]. Several cytogenetic nomenclatures for chromosome identification in *Avena* species were presented based on C-banding and FISH results, [16,49,54,55]. At the same time, their results were often contradictory and difficult to compare. Recently, a universal system for oat chromosome identification, based on multicolor FISH (MC-FISH) with a combination of several oligonucleotide probes and also sequential FISH painting with bulked oligoes specific to the wheat-barley linkage groups, was established [53]. At the same time, the level of genetic polymorphism and karyotype structures of interspecific hybrids of *A. sativa* and *A. macrostachya* still remain unstudied.

In the present study, based on marker sequences of satDNAs and the 18S–ITS1–5.8S rDNA region, we examined genome compositions and ribotypes in seven promising artificial fertile hybrids of *A. sativa* × *A. macrostachya* and also their ancestral species, *A. sativa*, and *A. macrostachya*, in order to clarify their ploidy status, as well as study chromosomal and genomic variability, which might accompany interspecific hybridization of polyploids.

## 2. Results

### 2.1. Chromosomal Structural Variations

**Figure 1:**
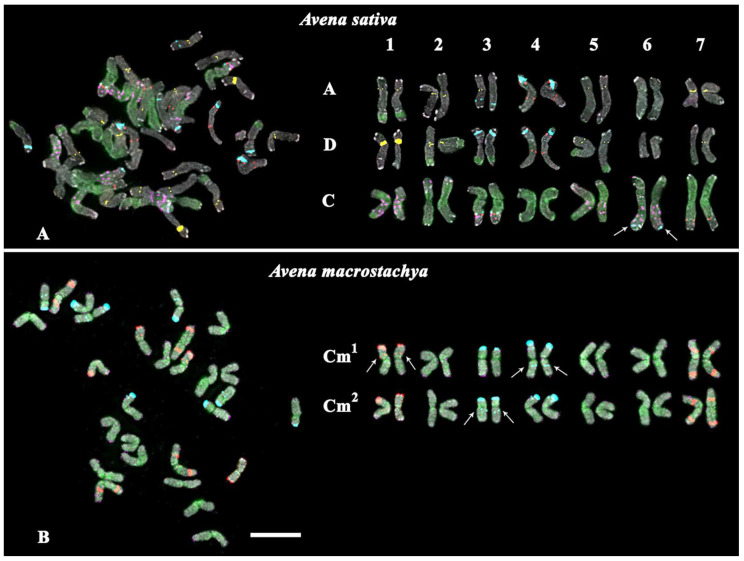
FISH-based localization of 35S rDNA (aqua), 5S rDNA (red), 6C343 (purple), 6C51 (green), and GTT (yellow) signals on chromosomes of (**A**) *Avena sativa* (subgenomes A, D, and C; arrows indicate the polymorphic minor 35S loci) and (**B**) *Avena macrostachya* (subgenomes Cm^1^ and Cm^2^; arrows indicate the minor 35S and 5S rDNA loci, which were not observed on the corresponding homoeologous chromosomes. DAPI-staining—grey. Scale bar—5 µm.

**Figure 2:**
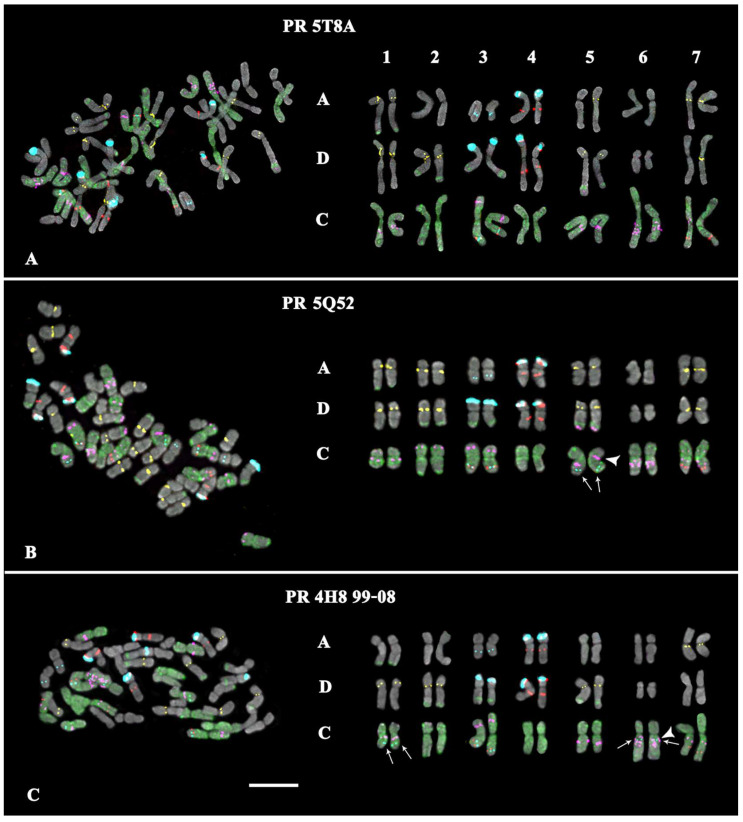
FISH-based localization of 35S rDNA (aqua), 5S rDNA (red), 6C343 (purple), 6C51 (green), and GTT (yellow) signals on chromosomes of the hexaploid (subgenomes A, D, and C) hybrids (**A**) PR 5T 8A, (**B**) PR 5Q52, and (**C**) PR 4H8 99-08. DAPI-staining—grey. Arrows point to the polymorphic minor 35S rDNA loci. The arrow head indicates chromosomal inversions. Scale bar—5 µm.

**Figure 3:**
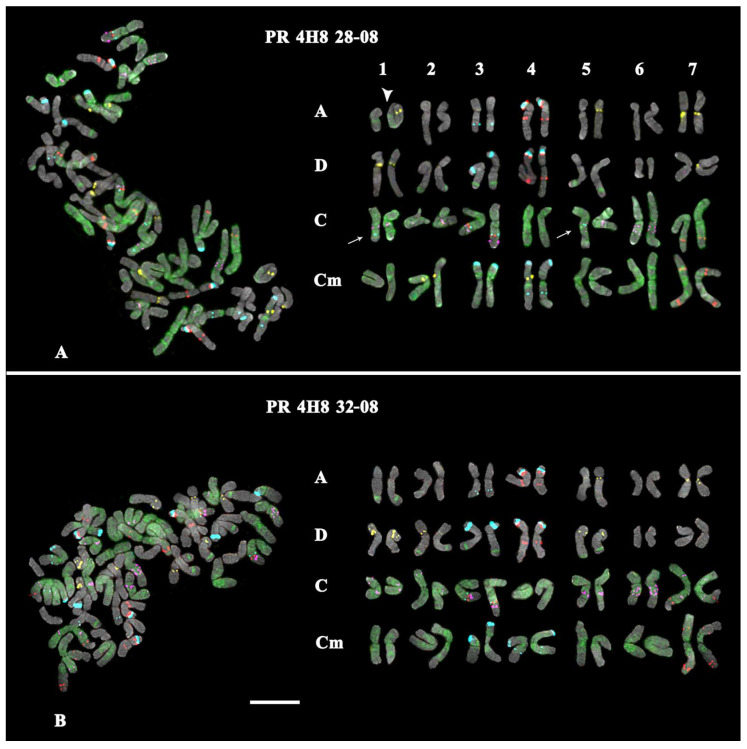
FISH-based localization of 35S rDNA (aqua), 5S rDNA (red), 6C343 (purple), 6C51 (green), and GTT (yellow) signals on chromosomes of the octoploid (subgenomes A, D, C, and Cm) hybrids (**A**) PR 4H8 28-08 and (**B**) PR 4H8 32-08. DAPI-staining—grey. Arrows point to the polymorphic minor 35S rDNA loci. The arrow head indicates a chromosomal translocation. Scale bar—5 µm.

**Figure 4:**
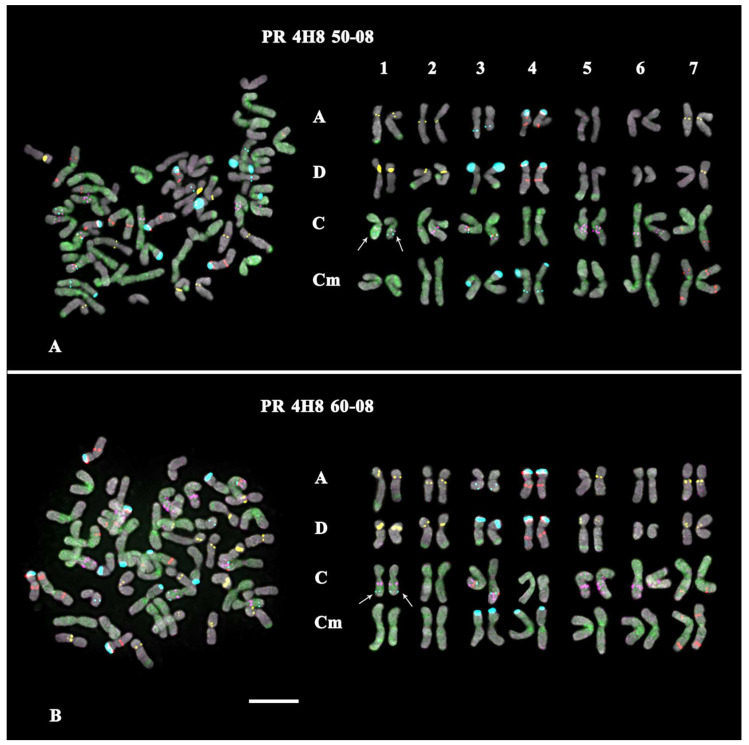
FISH-based localization of 35S rDNA (aqua), 5S rDNA (red), 6C343 (purple), 6C51 (green), and GTT (yellow) signals on chromosomes of the octoploid (subgenomes A, D, C, and Cm) hybrids (**A**) PR 4H8 50-08 and (**B**) PR 4H8 60-08. Arrows point to the polymorphic minor 35S rDNA loci. DAPI-staining—grey. Scale bar—5 µm.

Karyotypes of *A. sativa* and *A. macrostachya*, as well as seven artificial hybrids of *A. sativa* × *A. macrostachya*, were studied by MC-FISH, with a combination of five labelled probes (35S rDNA, 5S rDNA, oligo-(GTT)_10_, oligo-6C343, and oligo-6C51). In the studied karyotypes, all homologous chromosomes were identified, their subgenomic affiliation was determined, and chromosome karyograms and idiograms were constructed (Figure 1, Figure 2, Figure 3 and Figure 4; Supplementary Figures S1–S6).

The studied specimen of *A. sativa* had a hexaploid (ACD) karyotype with 2n = 6x = 42 chromosomes (Figure 1A and Supplementary Figure S1A). Large 35S rDNA clusters were localized in the subterminal regions of the short arms of chromosome pairs 4A, 3D, and 4D. In addition, constant minor 35S rDNA loci were identified in the intercalary regions of the long arms of chromosome pairs 3A and 3C. In addition, a polymorphic minor 35S rDNA cluster was detected in the distal regions of the long arms of chromosome pair 6C. Clusters of 5S rDNA were localized on chromosomes 4A and 4D (in the short arms in co-localization with 35S rDNA, as well as in the intercalary regions of the long arms), 3C (in the distal regions of the long arms), and 7C (in the distal regions). GTT clusters of varying intensity were detected in the pericentromeric regions of chromosome pairs 1A, 2A, 3A (the locus on pair 3A was in the hemizygous state), 5A, 7A, 1D, 2D, 5D, and 7D (the locus on pair 7D was in the hemizygous state). Clusters of 6C343 were observed on chromosome pairs 1C (multiple, in both arms), 2C (short arms, in the distal regions), 3C (long arms, in the distal regions), 5C (long arms, in the intercalary regions), and 6C (multiple, in both arms). Signals of 6C51 were dispersed along the chromosomes of the C subgenome, as well as localized in the distal regions of the long arms of chromosome pairs 1A, 2D, 3D, and 5D (Figure 1A; Supplementary Figures S1 and S5).

The studied specimen of *A. macrostachya* had a tetraploid karyotype (2n = 4x = 28) that was represented by two rather similar subgenomes (indicated as Cm^1^ and Cm^2^), which differed from each other only in distribution of minor 5S (chromosome pair 1) and 35S rDNA (chromosome pairs 3 and 4) loci (Figure 1B). In both Cm^1^ and Cm^2^ subgenomes, major 35S rDNA clusters were observed in the terminal regions of the short arms of chromosome pairs 3 and 4. Minor 35S rDNA loci were detected in the long arms of chromosome pair 3 (subgenome Cm^2^) and 4 (subgenome Cm^1^). In both subgenomes, minor 35S rDNA loci were also revealed in the distal region of the short arms of chromosome pair 4. Large 5S rDNA clusters were observed in the terminal regions of the short arms of chromosome pair 1, and also in the intercalary regions of the short arms and the distal regions of the long arms of chromosome pair 7. In one of the two subgenomes, minor 5S rDNA loci were revealed in the intercalary regions of the short arms of chromosome pair 1. Multiple hybridization signals of 6C51 were dispersed along all chromosomes of both Cm subgenomes. Only very small nonspecific signals of 6C343 were observed on *A. macrostachya* chromosomes (Figure 1B and Supplementary Figures S1 and S6).

The analysis of karyotypes of the studied interspecific hybrids *A. sativa* × *A. macrostachya* showed that the specimens of PR 5T 8A, PR 5Q52, and PR 4H8 99-08 were hexaploids (ACD, 2n = 6x = 42). Their karyotypes were similar to the *A. sativa* karyotype according to the chromosomal morphology and distribution of major clusters of the studied chromosomal markers (5S rDNA, 35S rDNA, 6C343, and 6C51). The remaining hybrids (PR 4H8 28-08, PR 4H8 32-08, PR 4H8 50-08, and PR 4H8 60-08) had octoploid karyotypes (ACCmD, 2n = 8x = 56), which were rather similar to corresponding chromosomes of *A. sativa* and *A. macrostachya* in chromosome morphology and distribution patterns of the chromosomal markers (Figure 1, Figure 2, Figure 3 and Figure 4 and Supplementary Figures S1–S6).

At the same time, some differences in patterns of chromosomal distribution of the studied markers were also observed. In particular, in the karyotypes of octoploid hybrids, any 5S rDNA clusters were not detected on chromosome pair 1Cm although they were observed in the *A. macrostachya* genome (Figure 1B, Figure 3, and Figure 4; Supplementary Figures S1, S3, S4, and S6). In all studied hybrids, constant minor 35S rDNA loci were observed on chromosome pairs 3A and 3C. However, variability in chromosome localization of polymorphic minor 35S rDNA and GTT loci was revealed:

- In the karyotype of PR 5T8A, only constant minor 35S rDNA loci were detected (on chromosome pairs 3A and 3C). On chromosome pairs 2A and 3A, no GTT clusters were revealed, although they were detected in *A. sativa*; and in pairs 5A and 7D, GTT loci were observed in the hemizygous state (Figure 2A; Supplementary Figures S2 and S5).
- In the karyotype of PR 5Q52, in addition to the constant minor 35S rDNA signals, a polymorphic minor locus was revealed on chromosome pairs 5C. A pericentric inversion occurred in one of the homologs of chromosome pair 5C. On chromosome pair 3A, a GTT locus was not detected (Figure 2B; Supplementary Figures S2 and S5).
- In the karyotype of PR 4H8 99-08, polymorphic minor 35S rDNA loci were revealed on chromosome pairs 1C and 6C. A chromosome inversion occurred within the long arms of chromosome pair 5C. On chromosome pairs 1A, 2A, 3A, 5A, and 7A, no GTT loci were observed (Figure 2C; Supplementary Figures S2 and S5).
- In the karyotype of PR 4H8 28-08, polymorphic minor 35S rDNA loci were detected on chromosome pairs 1C and 5C (both in the hemizygous state). In pair 1A, a translocation occurred between homologous chromosomes (Figure 3A). No GTT clusters were observed on chromosome pairs 2A, 3A, and 2D. Additional GTT clusters were revealed on chromosome pairs 2Cm and 4Cm (in the hemizygous state), which were not observed in the Cm genome of *A. macrostachya* (Figure 1B and Figure 3A; Supplementary Figures S3 and S6).
- In the karyotype of PR 4H8 32-08, only constant minor 35S rDNA loci were detected (on chromosome pairs 3A and 3C). On chromosome pairs 1A, 2A, 3A, and 7D, no GTT clusters were revealed (Figure 3B; Supplementary Figures S3 and S6).
- In the karyotype of PR 4H8 50-08, one polymorphic minor 35S rDNA locus was revealed on chromosome pair 1C. On chromosome pairs 3A, 5A, and 5D, no GTT signals were revealed; and in pair 7D, GTT clusters were observed in the hemizygous state (Figure 4A; Supplementary Figures S4 and S6).
- In the karyotype of PR 4H8 60-08, one polymorphic minor 35S rDNA locus was revealed on chromosome pair 1C. GTT signals were not revealed on chromosome pair 3A; and in pair 5A, GTT clusters were observed in the hemizygous state (Figure 4B; Supplementary Figures S4 and S6).

### 2.2. Molecular Phylogenetic Analysis

**Figure 5:**
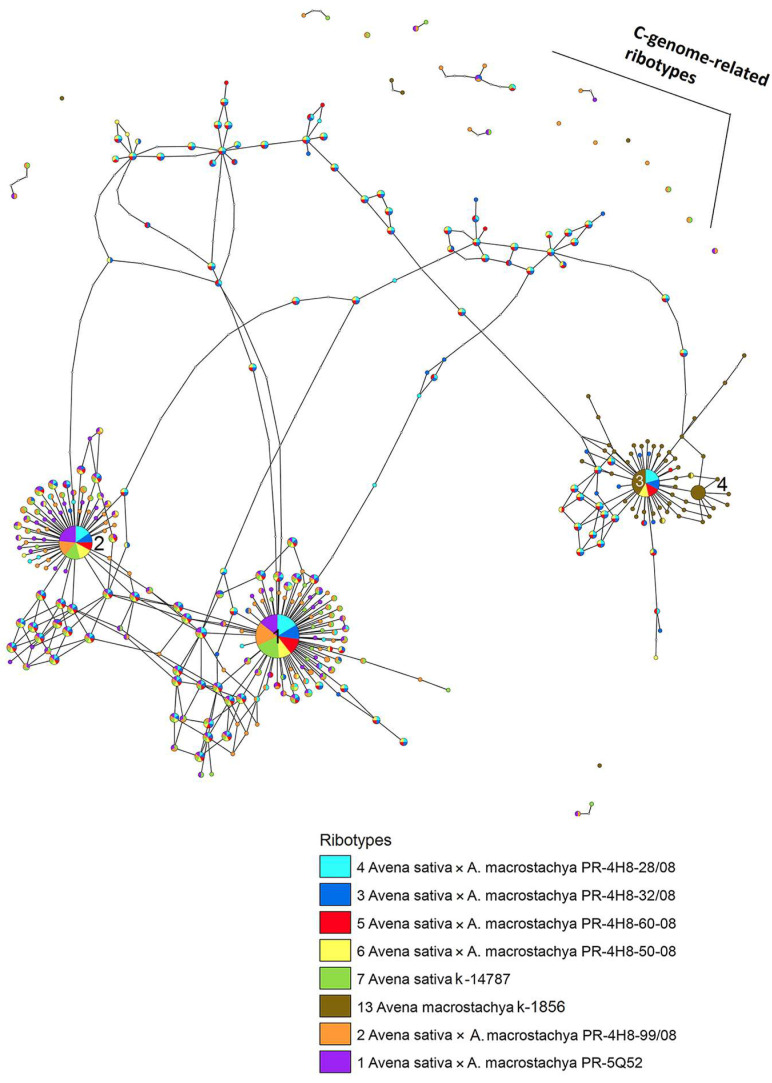
The ribotype network of the studied interspecific hybrids *Avena sativa* × *Avena macrostachya* and their parental species. The radius of each circle is proportional to the percent number of reads for each ribotype. Four major ribotypes (more than 1000 reads per rDNA pool) are marked with numbers. Small circles correspond to other ITS1 variants (less than 1000 reads per rDNA pool).

The aligned marker sequences (18S–ITS15.8S rDNA) of the studied specimens were sorted into ribotypes with a certain number of reads. We considered ribotypes with more than 1000 reads in the rDNA pool as major ones. To clear the relationships between the studied hybrids and their parental species, the ribotype network was developed by the method of statistical parsimony (Figure 5). The radius of the circles on this network was proportional to the percent number of reads for each ribotype. According to the ribotype network, four major ribotypes were identified in the rDNA pools of the studied specimens. They corresponded to some subgenomes of *A. sativa* (D or A) and *A. macrostachya* (Cm). C-genome-related ribotypes in ACD-hexaploids and the ACCmD-octoploids were represented only in minor fractions. They are shown as scattered dots in Figure 5.

The octoploid hybrids (PR 4H8 28-08, PR 4H8 32-08, PR 4H8 50-08, and PR 4H8 60-08) had three major ribotypes in their rDNA pool. The first major ribotype was the most frequent in terms of the quantity of reads (from 4346 reads, 18% in PR 4H8 28-08, to 3499 reads, 15% in PR 4H8 50-08) (marked by number 1 in Figure 5). This ribotype was clearly A-subgenome-related and probably represented the Dsubgenome of the ACD-hexaploids. The other two major ribotypes of the octoploid hybrids belonged to the A- and Cm subgenomes (Figure 5, marked by numbers 2 and 3, respectively). A-subgenome sequences of the octoploids were either the second (2196 reads, 9% in PR 4H8 32-08; 2881 reads, 12% in PR 4H8 50-08) or the third (1780 reads, 8% in PR 4H8 60-08) by quantity of reads. The PR 4H8 28-08 hybrid had almost the same quantity of reads in both A (1780 reads, 7%) and Cm (1785 reads, 8%) subgenomes.

The third major ribotype of the octoploid hybrids belonged to the Cmsubgenome, and this ribotype was common with one of the main ribotypes of *A. macrostachya* (Cm, 4971 reads, 26%). The second major ribotype of *A. macrostachya* (marked by number 4 in Figure 5) turned out to be species-specific, and it was not found in any rDNA pool of the studied hybrids (marked by number 4 in Figure 5).

The studied hexaploid hybrids (PR 5Q52 and PR 4H8 99-08) had two major A-genome-related ribotypes in their rDNA pool (Figure 5). One of them was common with the first major ribotype of the octoploid hybrids and *A. sativa* (9545 reads, 27% in PR 4H8 99-08 and 7892 reads, 22% in PR 5Q52). The second major ribotype of the hexaploid hybrids was shared with the second major ribotype of octoploid hybrids and *A. sativa* (Figure 5).

**Figure 6:**
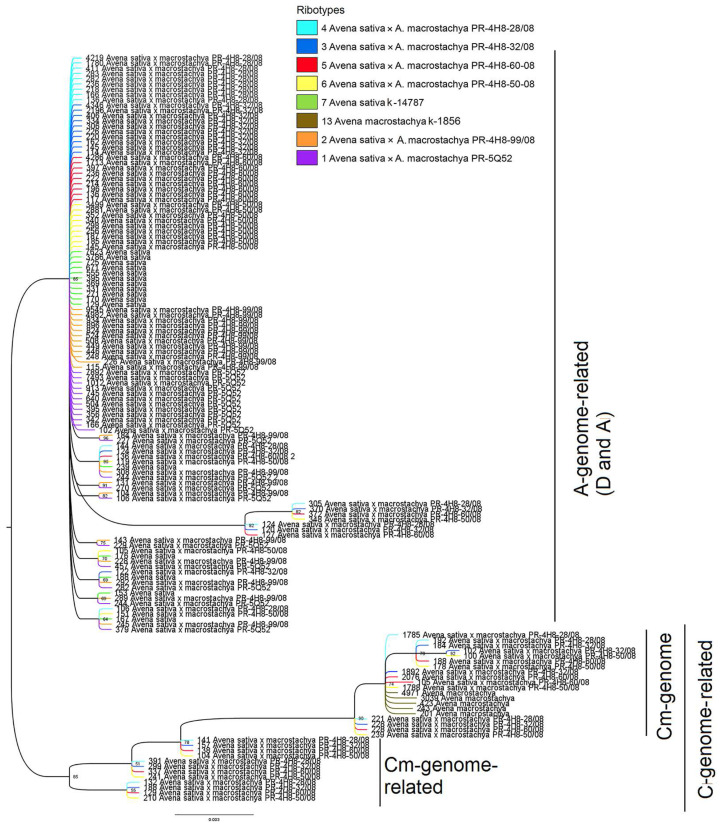
The ribotype-based phylogenetic tree, showing relationships among the studied interspecific hybrids *Avena sativa* × *Avena macrostachya* and their parental species. The index on the branch is the posterior probability in Bayesian inference. Numbers before the species name indicate the number of reads per the rDNA pool.

Based on the ribotypes, a phylogenetic tree showing relationships among interspecific hybrids *A. sativa* × *A. macrostachya* and their parental species was constructed by the Bayesian method. The phylogenetic tree demonstrates two main clades, which correspond to A- and C-genome-related ribotypes. Cm-related ribotypes of *A. macrostachya* form the separate clade within the C-genome clade and do not differ from Cmribotypes of the octoploid hybrids (Figure 6).

## 3. Discussion

Hybridization plays a fundamental role in plant evolution and breeding, as it can result in phenotypic changes, sexual isolation, and the appearance of new species [56]. In spontaneous and artificial hybridization, the merging of two or more different genomes can often be accompanied by a phenomenon called a ‘genomic shock’, which leads to a wide genetic and epigenetic changes in a hybrid [57,58]. Interspecific hybridization is widely used in crop breeding since it expands the species variability that is necessary for emergence of new polyploid hybrids with improved characteristics compared to their parental species [59,60]. In *Avena* breeding programs, for example, the interspecific crosses with wild relatives, such as winter-hardy *A. macrostachya*, were used to produce new cold-resistant allopolyploid hybrid cultivars [36,38,39,40]. A comparative study of genomes of artificial and natural allopolyploids contributes to understanding the pattern of formation of a new hybrid genome after the ‘genomic shock’. In the present study, the performed FISH-based karyogenomic studies of seven promising interspecific hybrids *A. sativa* × *A. macrostachya* revealed several chromosomal structural rearrangements in karyotypes of three hybrids, which could also be related to the post-hybridization genomic instability.

Repetitive DNA sequences constitute a significant component (from 25% to 85%) of the genome of most plants. DNA repeats can play a crucial role in the speciation since their motifs can vary greatly in sequence and dispersion patterns [16,52,61,62]. The repeatome is considered to play important roles in the eukaryotic genome; for example, it can be involved in genome stability, recombination, chromatin modulation, and the modification of gene expression [62]. Major groups of plant DNA repeats include 35S (18S–5.8S–26S) and 5S rDNAs with variable intergenic spacers, transposable elements, and also satellite DNAs, which are highly abundant and diverse parts of genomes [52,62,63]. These repeats, as well as microsatellite DNAs, are often used as probes for FISH analyses to investigate the genetic diversity in *Avena* species, since they can generate specific patterns of FISH signal distribution on individual chromosomes [48,49,50,64]. Recently, a system of FISH painting with bulked satDNA oligo-probes, based on wheat–barley collinear regions, was designed to validate the linkage group assignment for individual *A. sativa* chromosomes [53]. MC-FISH with the use of a combination of different labelled marker satDNAs generates chromosome- and genome-specific patterns, and consequently, it could be a valuable tool for studying processes of structural and molecular reorganization occurring in allopolyploid genomes after hybridization. In the present study, to examine the genetic diversity, identify possible chromosomal rearrangements, and clarify evolutionary relationships among the studied *Avena* specimens, we used MC-FISH with a combination of various labelled probes, namely chromosome-specific oligo-6C343 and C-genome-specific oligo-6C51 from the system of Jiang et al. [53]; one microsatellite marker (oligo-(GTT)_10_) previously used in *Avena* chromosome analyses [50]; and also two classical chromosome markers, 5S and 35S rDNA, which were studied earlier in other specimens of parental *A. sativa* and *A. macrostachya* [48,64]. In our study, this set of chromosome markers turned out to be optimal to identify individual chromosomes, as well as clarify their subgenomic affiliation in karyotypes, which facilitated comparative cytogenomic analyses in the studied plants. In addition, this approach allows us to compare the karyotypes of *A. sativa* and *A. macrostachya* with previously studied specimens of these species [48,50,53,64].

The 35S rRNA genes encoding 18S, 5.8S, and 26S rRNA are essential constituents of all eukaryotic genomes [65,66]. Plant genomes bear a high number (from 200 to 22,000) of the 35S rRNA genes per a haploid genome, and these genes are arranged in tandem arrays and localized on one or several chromosomes [66,67]. For example, in the A-genome diploid species of *Avena*, two to three 35S rDNA clusters per haploid chromosome set were detected [48,52,68,69]. According to previous molecular phylogenetic studies of *Avena* species, two NORs (major 35S rDNA loci) per haploid chromosome set were ancestral characters, and four or more NORs were derived characters [70]. In hexaploid *A. sativa*, however, the elimination of major rDNAs in the Csubgenome and partial elimination of rDNAs in the Asubgenome were revealed, indicating that rDNA from one ancestor (probably, from the paternal genome) might be silenced or lost after hexaploidization [12,52]. In consistency with these data, we did not reveal major 35S rDNA clusters on C-subgenome chromosomes of *A. sativa*. At the same time, we detected six minor 35S rDNA loci localized on two chromosome pairs of Csubgenome and one pair of Asubgenome. It was less than the number of the minor 35S rDNA loci (eight) reported earlier for another *A. sativa* specimen [64], which indicated the presence of chromosomal diversity among different specimens of *A. sativa*.

Moreover, we detected differences in chromosome distribution patterns of minor 35S rDNA loci among the resulting hybrids and their parental species. At the same time, major 35S rDNA clusters that belonged to A and Dsubgenomes were not eliminated in the karyotypes of the studied hexaploid or octoploid hybrids, indicating that the loss of 35S rRNA genes in genomes of allopolyploids could be a rather long and ambiguous process, in contrast to their inactivation (nucleolar dominance) [71,72].

In karyotype of *A. macrostachya*, we observed two rather similar Cmsubgenomes differing; however, this was in the chromosome localization of minor loci of 5S and 35S rDNA, which allows us to designate them as Cm^1^ and Cm^2^. This could be related to its allopolyploid nature, although previous studies classified this species as an autotetraploid [32,41,48]. Theoretically, the genomic composition of newly formed interspecific hybrids *A. sativa* (ACD) × *A. macrostachya* (Cm^1^Cm^2^) could be represented by complex allopolyploid genome ACCm^1^Cm^2^D. However, only the hybrids with the maternal genomic composition and also the hybrids containing the whole maternal genome and one of the paternal subgenomes remained viable. It also turned out that all octoploid hybrids lacked chromosome pair 1Cm with 5S rDNA clusters in the short arms, which was presented in both Cmsubgenomes of *A. macrostachya*. In the resulting hybrids, these 5S rDNA clusters could be deleted since structural variations as well as asymmetric divergence of subgenomes have already been found in other plant species after allopolyploidization [73]. Anyway, our results show that both 35S rDNA and 5S rDNA are involved in the process of formation of a new allopolyploid genome.

We observed polymorphic localization of (GTT)_10_ clusters on chromosomes of the studied hybrids. Moreover, in one octoploid (PR 4H8 28-08), (GTT)_10_ clusters were even detected on chromosomes of the Cmsubgenome, although this microsatellite marker was not revealed in the paternal *A. macrostachya* karyotype or on chromosomes of the Csubgenome in maternal *A. sativa*. The C subgenome of hexaploid oats is highly diverged from the A and Dsubgenomes, which was demonstrated by C-banding [46,74] and FISH analyses [27,75,76,77]. In this research, the unusual localization of (GTT)_10_ clusters on chromosomes of the Cmsubgenome could be the result of rearrangements in chromosomes of parental genomes occurred after polyploidization. It was previously shown that FISH-based patterns of chromosome distribution of some microsatellites, including GTT motifs, can be highly polymorphic [50]. At the same time, in karyotypes of the studied specimens, we observed constant localization of (GTT)_10_ clusters in the pericentromeric regions of chromosome pairs 7A and 1D, which could be used in further studies as an additional chromosomal marker that facilitate the subgenomic affiliation of oat chromosomes.

The molecular phylogenetic examination of intragenomic rDNA polymorphism is successfully used to study the origin and relationships among *Avena* species [78,79,80]. It was previously shown that in some cases the ITS1 region might be a more effective marker than ITS2 [81] due to its conservative structure and higher substitution rate [82,83,84]. We also used the ITS1–5.8S rDNA region as a marker sequence to study intragenomic rDNA variability among the allopolyploid hybrids and their parental species, since this approach is suitable for identifying hidden polymorphism in hybrid taxa in cases of multiple hybridization [85,86].

In the present study, the first major ribotype was the most frequent in the rDNA pool. It was clearly A-genome-related and probably represented the Dsubgenome of the ACD-hexaploids including *A. sativa* according to our previous studies [21,80]. The second major ribotype was also A-genome-related and probably belonged to the Asubgenome [21,80]. These data are consistent with our molecular cytogenetic results, which revealed two major 35S rDNA clusters in the Dsubgenome and one major 35S rDNA cluster in the Asubgenome in the studied karyotypes of hexaploids and octoploids. Distant hybridization could lead to a redistribution of rDNA between subgenomes in the resulting allopolyploid; for example, our previous molecular studies showed that major ribotypes, which presented in C-genome diploid *Avena* species, were mainly eliminated from the rDNA pool of various *Avena* hexaploids [21,80]. In the present study, C-subgenome-related ribotypes were also presented only in minor fractions in the rDNA pools of the studied *A. sativa* and hybrids. Our molecular cytogenetic analysis confirmed these results since only minor 35S rDNA loci were visualized on C-subgenome chromosomes.

As mentioned above, we revealed differences in chromosome distribution of minor 35S rDNA loci between two Cmsubgenomes of *A. macrostachya*, which could be related to its allopolyploid nature. Moreover, we found that only one of the Cmsubgenomes of *A. macrostachya* was predominantly inherited in octoploid hybrids. According to our molecular data, the third major ribotype was shared with the octoploid hybrids and *A. macrostachya*, which indicated that it could represent one of the Cmsubgenomes of *A. macrostachya*. The fourth major ribotype turned out to be species-specific and it was not found in rDNA pools of the studied hybrids. This ribotype probably represented the second Cmsubgenome of *A. macrostachya*, which also supported the assumption of an allopolyploid origin of this species.

Our phylogenetic tree confirms the previous statements about the close relationship between A and Dgenomes [14,27,75] as well as their significant distance from the C genome [11,14]. In addition, our tree clearly supports the hypothesis of C-genome nature of *A. macrostachya* [40]. *A. macrostachya* is thought to be the most primitive within oats [17] but nevertheless it can be close to the ancestral species of modern C-genome *Avena* diploids [21].

Thus, our findings indicate that the use of marker sequences of satDNAs and the ITS1–5.8S rDNA region in comprehensive cytogenomic and molecular studies can provide novel important data on genomic relationships within *Avena* allopolyploid species and hybrids, as well as expand the potential for interspecific crosses for breeding.

## 4. Materials and Methods

### 4.1. Plant Material

Seeds of *Avena sativa* (k-11840, cultivar Borrus, Germany; k-14787 cultivar Privet, Russia), *A. macrostachya* (k-1856, Algeria) and seven artificial stable fertile hybrids *A. sativa* (ACD) × *A. macrostachya* (Cm), Poland, namely PR 4H8 28-08 (pr.k-4528), PR 4H8 32-08 (pr.k-4529), PR 4H8 99-08 (pr.k-4532), PR 4H8 60-08 (pr.k-4531), PR 4H8 50-08 (pr.k-4530), PR 5T8A (k-15711), and PR 5Q52 (k-15712), were obtained from the seed collection of FRC N.I. Vavilov All-Russian Institute of Plant Genetic Resources (VIR).

### 4.2. Chromosome Spread Preparation

The chromosome spread preparations were made as described previously with minor modifications [87]. The seeds were germinated (usually, for 3–5 days) at room temperature (RT) in Petri dishes using moist filter paper. Root tips (0.5–1 cm long) were excised and placed into ice-cold water for 16–20 h for accumulation of mitotic divisions. Then, the roots were fixed in the ethanol/acetic acid (3:1) fixative for 48 h at room temperature. The fixed roots were transferred into 1% acetocarmine solution in 45% acetic acid for 30 min. Then, each root was placed on the slide, the root meristem was cut from the root cap, and a squashed preparation was made using a cover slip. After freezing in liquid nitrogen and removing the cover slip, the slide was dehydrated in 96% ethanol and air dried.

### 4.3. Multicolor Fluorescence In Situ Hybridization

For sequential MC-FISH assays, we used a combination of five labelled DNA probes: (1) pTa71 enclosing the 18S–5.8S–26S (35S) rDNA sequence of common wheat [88]; (2) pTa794 containing the 5S rDNA sequence of common wheat [89]; and three sequences of oligonucleotide DNA probes, namely (3) oligo-(GTT)_10_; (4) oligo-6C343 (AGGACATATGTACATGGAGAGCCAAGGTTGGGCCAACTTTGCCACATTCT) [53]; and (5) oligo-6C51 (AACACACATGCAATGACTCTAGTGGTTGATCCATGTGTGGTTTGTGGAAAG) [53].

Both pTa71 and pTa794 were labelled directly with fluorochromes Aqua 431 dUTP and Red 580 dUTP (ENZO Life Sciences, Farmingdale, NY, USA) by nick translation according to the manufacturer’s protocols. The oligo-(GTT)_10_ probe was synthesized using a synthesizer ABI 394 (applied BioSystems, Redwood City, CA, USA) and labelled at the 3′-end with fluorescein-12-dUTP (Roche diagnostics, Mannheim, Germany). Both oligo-6C51 and oligo-6C343 were produced and labelled directly with FAM or ROX fluorochromes in *Syntol* (Moscow, Russia).

Several sequential FISH procedures were performed with various combinations of these labelled DNA probes as described previously [87]. Before the first FISH procedure, chromosome slides were pretreated with RNAse A (Roche Diagnostics, Mannheim, Germany) dissolved in 2 × SSC (1 mg/mL) for 1 h at 37 °C, washed three times (for 10 min each) in 2 × SSC, dehydrated through a graded ethanol series (70%, 85%, and 96%) for 2 min each and air dried. Then, 40 ng of each labelled probe was dissolved in hybridization mixture (50% formamide, 70% of hybridization specificity (stringency)) in a total volume of 15 µL and dropped to each slide. Afterwards, the slides were covered with a coverslip, sealed with rubber cement, denatured at 74 °C for 5 min, chilled on ice, and placed in a moisture chamber at 37 °C. After overnight hybridization, the slides were washed in 0.1 × SSC (8 min, 42 °C) and twice in 2 × SSC (8 min at 42 °C), followed by a 5 min wash in 2 × SSC and two 3 min washes in PBS at RT. Then, the slides were dehydrated, air dried and stained with DAPI (4′,6-diamidino-2-phenylindole) dissolved (0.1 µg/mL) in Vectashield mounting medium (Vector Laboratories, Burlingame, CA, USA). After documenting FISH results, the chromosome slides were washed twice in distilled water for 5 min. Then, sequential FISH procedures were conducted on the same slides.

### 4.4. Chromosome Analysis

Chromosome slides were analyzed using the Olympus BX-61 epifluorescence microscope (Olympus, Tokyo, Japan). Chromosome images were captured with monochrome charge-coupled device camera (Cool Snap, Roper Scientific, Inc., Tucson, AZ, USA). Then, the images were pseudo-colored and processed using Adobe Photoshop 10.0 (Adobe, Birmingham, CA, USA) software. At least 5 plants (15 metaphase plates for each plant) were analyzed. Chromosome pairs in karyotypes were identified according to the chromosome size and morphology, as well as localization of the studied chromosome markers. The chromosome identification and subgenome affiliation of *A. sativa* were performed according to the classification of Jiang et al. [53]. In *A. macrostachya* karyograms, the chromosomes bearing the signal localization variants observed in karyotypes of the octoploid hybrids, were placed in one of the two subgenomes (Cm^1^). In karyograms of *A. macrostachya* and octoploid hybrids, Cm chromosome pairs were set in the increasing order of size.

### 4.5. Molecular Phylogenetic Analysis

Genomic DNA was extracted from dried leaves and seeds of the studied hybrids and their parental species using the Qiagen Plant Mini Kit (Qiagen, Hilden, Germany), according to the manufacturer’s recommendations. The sequences of 18S–ITS1–5.8S rDNA were obtained via NGS (the Illumina MiSeq Platform) at the Shared Use Center ‘Genomic Technologies, Proteomics, and Cell Biology’ of the All-Russian Research Institute of Agricultural Microbiology (Pushkin, St. Petersburg, Russia).

PCR was carried out in 15 μL of the reaction mixture containing 0.5–1 unit of activity of Q5 High-Fidelity DNA Polymerase (NEB, Ipswich, MA, USA), 5 pM of forward and reverse primers, 10 ng of DNA template, and 2 nM of each dNTP (Life Technologies, ThermoScientific, Waltham, MA, USA). It was amplified as follows: initial denaturation at 94 °C for 1 min; then 25 cycles of 94 °C for 30 s, 55 °C for 30 s, and 72 °C for 30 s; and then a final elongation at 72 °C for 5 min, using ITS 1P (AACCTTATCATTTAGAGGAAGG) [90] and ITS 2 (GCTGCGTTCTTCATCGATGC) [91] primers. The resulting marker fragments ranged in length from 302 to 344 base pairs and included the 18S rDNA-ITS1-5.8S rDNA region. PCR products were purified according to the Illumina recommended technique using AMPureXP (Beckman Coulter, Indianapolis, IN, USA). The libraries were prepared according to the manufacturer’s MiSeq Reagent Kit Preparation Guide (Illumina, San Diego, CA, USA) (http://web.uri.edu/gsc/files/16s-metagenomic-library-prep-guide-15044223-b.pdf (accessed on 11 May 2023)). Then, they were sequenced with the Illumina MiSeq system (Illumina, San Diego, CA, USA) using a MiSeq Reagent Kit v3 (600 cycles) with double-sided reading (2 × 300 n) according to the manufacturer’s instructions. The raw sequencing data for *Avena sativa* (PP314881–PP314972), *A. macrostachya* (PP314828–PP314880), and six hybrids *Avena sativa* × *A. macrostachya*, namely PR 4H8 28-08 (PP314355–PP314468), PR 4H8 32-08 (PP314469–PP314597), PR 4H8 60-08 (PP314598–PP314710), PR 4H8 50-08 (PP314711–PP314827), PR4H8 99-08 (PP314973–PP315106), and PR 5Q52 (PP315107–PP315236) were uploaded to the National Center for Biotechnology Information (NCBI) BioProject database (https://submit.ncbi.nlm.nih.gov/subs/genbank/SUB14212443/overview, accessed on 18 April 2024). Additional sequences information for the ribotype-based phylogenetic tree and the ribotype network was included in the Supplementary Files S1 and S2.

These sequences were trimmed with Trimmomatic [92] included in Unipro Ugene [93] using the following parameters: PE reads, sliding window trimming with size 4, quality threshold 12, and minimal read length 130. The paired marker sequences were combined, dereplicated, and sorted into the ribotypes with the vsearch 2.7.1 tool [94]. The resulting sequences represented ribotypes in the whole pool of genomic rDNA, which were filtered based on their frequencies. Then, they were analyzed by statistical parsimony network (TCS software, version 1.21) [95] and visualized with the TCSBU program [96]. The threshold for this analysis was 10 reads per the whole genome pool. Moreover, we built phylogenetic trees of the ribotypes by the Bayesian inference and maximum likelihood method. The threshold for tree inference was 100 reads per the rDNA pool. We used the MrBayes program (version 3.2.2) for the Bayesian estimation of phylogeny [97], according to the GTR+G model previously estimated by MEGA XI [98]. The Bayesian analysis was conducted with 5–8 million generations, sampling trees every 100 generations, and the first 25% trees were discarded as burn-in. The obtained phylogenetic trees were visualized and edited in the FigTree (version 1.4.3) software (http://tree.bio.ed.ac.uk/, accessed 1 March 2023).

## Data Availability

All data generated or analyzed during this study are contained within the article and the Supplementary section.

## Supplementary Materials

The following supporting information can be downloaded at: https://www.mdpi.com/article/10.3390/ijms25105534/s1.

## References

[1] Strychar R., Webster F., Wood P. (2011). World Oat Production, Trade, and Usage. Oats: Chemistry and Technology.

[2] Zhang J., Li X., Wang J., Yang L., Yang Q., Xiang D., Wan Y., Nevo E., Yan J., Fan Y. (2023). Wild oats offer new possibilities for forage because of the higher nutrition content and feed value. Agronomy.

[3] Yan H., Martin S.L., Bekele W.A., Latta R.G., Diederichsen A., Peng Y., Tinker N.A. (2016). Genome size variation in the genus *Avena*. Genome.

[4] Peng Y., Yan H., Guo L., Deng C., Wang C., Wang Y., Kang L., Zhou P., Yu K., Dong X. (2022). Reference genome assemblies reveal the origin and evolution of allohexaploid oat. Nat. Genet..

[5] Murat F., Xu J.H., Tannier E., Abrouk M., Guilhot N., Pont C., Messing J., Salse J. (2010). Ancestral grass karyotype reconstruction unravels new mechanisms of genome shuffling as a source of plant evolution. Genome Res..

[6] Panchy N.L., Azodi C.B., Winship E.F., O’Malley R.C., Shiu S.H. (2019). Expression and regulatory asymmetry of retained Arabidopsis thaliana transcription factor genes derived from whole genome duplication. BMC Evol. Biol..

[7] Salse J. (2016). Deciphering the evolutionary interplay between subgenomes following polyploidy: A paleogenomics approach in grasses. Am. J. Bot..

[8] Liu Q., Ye L., Li M., Wang Z., Xiong G., Ye Y., Tu T., Schwarzacher T., Heslop-Harrison J.S. (2023). Genome-wide expansion and reorganization during grass evolution: From 30 Mb chromosomes in rice and *Brachypodium* to 550 Mb in *Avena*. BMC Plant Biol..

[9] Thomas H., Marshall H.G., Sorrells M.E. (1992). Cytogenetics of *Avena*. Oat Science and Technology.

[10] Katsiotis A., Loukas M., Heslop-Harrison J.S. (2000). Repetitive DNA, genome and species relationships in *Avena* and *Arrhenatherum* (Poaceae). Ann. Bot..

[11] Loskutov I.G. (2008). On evolutionary pathways of *Avena* species. Genet. Resour. Crop. Evol..

[12] Liu Q., Lin L., Zhou X., Peterson P.M., Wen J. (2017). Unraveling the evolutionary dynamics of ancient and recent polyploidization events in *Avena* (Poaceae). Sci. Rep..

[13] Fu Y.-B., Williams D.J. (2008). AFLP variation in 25 *Avena* species. Theor. Appl. Genet..

[14] Loskutov I., Rines H.W., Kole C. (2011). Avena. Wild Crop Relatives: Genomic and Breeding Resources, Cereals.

[15] Li W.T., Peng Y.Y., Wei Y.M., Baum B.R., Zheng Y.L. (2009). Relationship among *Avena* species as revealed by consensus chloroplast simple sequence repeat (ccSSR) markers. Genet. Resour. Crop. Evol..

[16] Tomas D., Rodrigues J., Varela A., Veloso M.M., Viegas W., Silva M. (2016). Use of repetitive sequences for molecular and cytogenetic characterization of *Avena* species from Portugal. Int. J. Mol. Sci..

[17] Rodionov A.V., Tiupa N.B., Kim E.S., Machs E.M., Loskutov I.G. (2005). Genomic structure of the autotetraploid oat species *Avena macrostachya* inferred from comparative analysis of the ITS1 and ITS2 sequences: On the oat karyotype evolution during the early stages of the *Avena* species divergence. Genetika.

[18] Nikoloudakis N., Skaracis G.A., Katsiotis A. (2008). Evolutionary insights inferred by molecular analysis of the ITS1-5.8S-ITS2 and IGS *Avena* sp. sequences. Mol. Phyl. Evol..

[19] Peng Y.Y., Wei Y.M., Baum B.R., Yan Z.H., Lan X.J., Dai S.F., Zheng Y.L. (2010). Phylogenetic inferences in *Avena* based on analysis of FL intron2 sequences. Theor. Appl. Genet..

[20] Rodrigues J., Viegas W., Silva M. (2017). 45S rDNA external transcribed spacer organization reveals new phylogenetic relationships in *Avena* genus. PLoS ONE.

[21] Gnutikov A.A., Nosov N.N., Loskutov I.G., Machs E.M., Blinova E.V., Probatova N.S., Langdon T., Rodionov A.V. (2022). New insights into the genomic structure of the oats (*Avena* L., Poaceae): Intragenomic polymorphism of ITS1 sequences of rare endemic species *Avena bruhnsiana* Gruner and its relationship to other species with C-genomes. Euphytica.

[22] Rajhathy T., Thomas H. (1974). Cytogenetics of Oats.

[23] Baum B.R. (1977). Oats: Wild and Cultivated. A Monograph of the Genus Avena L. (Poaceae).

[24] Fu Y.B. (2018). Oat evolution revealed in the maternal lineages of 25 *Avena* species. Sci. Rep..

[25] Irigoyen M., Loarce Y., Linares C., Ferrer E., Leggett M., Fominaya A. (2001). Discrimination of the closely related A and B genomes in AABB tetraploid species of *Avena*. Theor. Appl. Genet..

[26] Katsiotis A., Hagidimitriou M., Heslop-Harrison J.S. (1997). The close relationship between the A and B genomes in *Avena* L. (Poaceae) determined by molecular cytogenetic analysis of total genomic, tandemly and dispersed repetitive DNA sequences. Ann. Bot..

[27] Linares C., Ferrer E., Fominaya A. (1998). Discrimination of the closely related A and D genomes of the hexaploid oat *Avena sativa* L.. Proc. Natl. Acad. Sci. USA.

[28] Ananiev E.V., Vales M.I., Phillips R.L., Rines H.W. (2002). Isolation of A/D and C genome specific dispersed and clustered repetitive DNA sequences from *Avena sativa*. Genome.

[29] Kamal N., Tsardakas Renhuldt N., Bentzer J., Gundlach H., Haberer G., Juhász A., Lux T., Bose U., Tye-Din J.A., Lang D. (2022). The mosaic oat genome gives insights into a uniquely healthy cereal crop. Nature.

[30] Mark B., Andon J.W. (2008). State of the art reviews: The oatmeal-cholesterol connection: 10 years later. Am. J. Lifestyle Med..

[31] Agostoni C., Bresson J., Fairweather-Tait S., Flynn A., Golly I., Korhonen H., Lagiou P., Løvik M., Marchelli R., Martin A. (2010). Scientific opinion on the substantiation of a health claim related to oat beta glucan and lowering blood cholesterol and reduced risk of (coronary) heart disease pursuant to Article 14 of Regulation (EC) No 1924/2006. EFSA J..

[32] Baum B.R., Rajhathy T. (1976). A study of *Avena macrostachya*. Can. J. Bot..

[33] Bolc P., Łapiński B., Podyma W., Boczkowska M. (2020). Genetic diversity and population structure of Algerian endemic plant species *Avena macrostachya* Bal. ex Cross. et Durieu. Agronomy.

[34] Weibull J. (1988). Resistance in the wild crop relatives *Avena macrostachya* and *Hordeum bogdani* to the aphid *Rhopalosiphum padi*. Entomol. Exp. Appl..

[35] Yu J., Herrmann M. (2006). Inheritance and mapping of a powdery mildew resistance gene introgressed from *Avena macrostachya* in cultivated oat. Theor. Appl. Genet..

[36] Łapinski B., Kała M., Nakielna Z., Jellen R., Livingston D., Behl R.E.A. (2013). The perennial wild species *Avena macrostachya* as a genetic source for improvement of winterhardiness in winter oat for cultivation in Poland. Biotechnology and Plant Breeding—Perspectives.

[37] Hoppe H.-D., Pohler W. (1988). Successful hybridization between *Avena prostrata* and *Avena macrostachya*. Cereal Res. Comm..

[38] Jia H., Livingston D.P., Murphy J.P., Porter D.R. (2006). Evaluation of freezing tolerance in advanced progeny from a cross of *Avena sativa* × *Avena macrostachya*. Cereal Res. Commun..

[39] Lapinski B., Rachwalska A. (2017). Using *Avena macrostachya* for improvement of oat winterhardiness in Poland. Proc. Appl. Bot. Genet. Breed..

[40] Pohler W., Hoppe H.-D. (1991). Homeology between the chromosomes of *Avena macrostachya* and the *Avena* C genome. Plant Breed..

[41] Leggett J.M. (1992). Further hybrids involving the perennial autotetraploid oat *Avena macrostachya*. Genome.

[42] Cheng D.W., Armstrong K.C., Drouin G., McElroy A., Fedak G., Molnar S.D. (2003). Isolation and identification of *Triticeae* chromosome 1 receptor-like kinase genes (Lrk10) from diploid, tetraploid, and hexaploid species of the genus *Avena*. Genome.

[43] Peng Y.-Y., Wei Y.-M., Baum B.R., Zheng Y.-L. (2008). Molecular diversity of the 5S rRNA gene and genomic relationships in the genus *Avena* (Poaceae: Aveneae). Genome.

[44] Leggett J.M., Markhand G.S., Brandham P.E., Bennett M.D. (1995). The Genomic Configuration of Avena Revealed by GISH, Kew Chromosome Conf. IV.

[45] Postoyko J., Hutchinson J., Lawes D.A., Thomas H. (1986). The identification of *Avena* chromosomes by means of C-banding. Proceedings of the 2nd International Oat Conference, The University College of Wales, Welsh Plant Breeding Station.

[46] Fominaya A., Vega C., Ferrer E. (1988). Giemsa C-banded karyotypes of *Avena* species. Genome.

[47] Chen Q., Armstrong K. (1994). Genomic in situ hybridization in *Avena sativa*. Genome.

[48] Badaeva E.D., Shelukhina O.Y., Diederichsen A., Loskutov I.G., Pukhalskiy V.A. (2010). Comparative cytogenetic analysis of *Avena macrostachya* and diploid C-genome *Avena* species. Genome.

[49] Fominaya A., Loarce Y., Montes A., Ferrer E. (2017). Chromosomal distribution patterns of the (AC)_10_ microsatellite and other repetitive sequences, and their use in chromosome rearrangement analysis of species of the genus *Avena*. Genome.

[50] Luo X.M., Tinker N.A., Zhou Y.H., Liu J.C., Wan W.L., Chen L. (2018). Chromosomal distributions of oligo-Am1 and (TTG)_6_ trinucleotide and their utilization in genome association analysis of sixteen *Avena* species. Genet. Resour. Crop. Evol..

[51] Maughan P.J., Lee R., Walstead R., Vickerstaff R.J., Fogarty M.C., Brouwer C.R., Reid R.R., Jay J.J., Bekele W.A., Jackson E.W. (2019). Genomic insights from the first chromosome-scale assemblies of oat (*Avena* spp.) diploid species. BMC Biol..

[52] Liu Q., Li X., Zhou X., Li M., Zhang F., Schwarzacher T., Heslop-Harrison J.S. (2019). The repetitive DNA landscape in *Avena* (Poaceae): Chromosome and genome evolution defined by major repeat classes in whole-genome sequence reads. BMC Plant Biol..

[53] Jiang W., Jiang C., Yuan W., Zhang M., Fang Z., Li Y., Li G., Jia J., Yang Z. (2021). A universal karyotypic system for hexaploid and diploid *Avena* species brings oat cytogenetics into the genomics era. BMC Plant Biol..

[54] Sanz M.J., Jellen E.N., Loarce Y., Irigoyen M.L., Ferrer E., Fominaya A. (2010). A new chromosome nomenclature system for oat (*Avena sativa* L. and *A. byzantine* C. Koch) based on FISH analysis of monosomic lines. Theor Appl Genet.

[55] Chaffin A.S., Huang Y.F., Smith S., Bekele W.A., Babiker E., Gnanesh B.N., Foresman B.J., Blanchard S.G., Jay J.J., Reid R.W. (2016). A consensus map in cultivated hexaploid oat reveals conserved grass synteny with substantial subgenome rearrangement. Plant Genome.

[56] Mason A.S., Batley J. (2015). Creating new interspecific hybrid and polyploid crops. Trends Biotechnol.

[57] Comai L., Madlung A., Josefsson C., Tyagi A. (2003). Do the different parental ‘heteromes’ cause genomic shock in newly formed allopolyploids?. Philos. Trans. R. Soc. B Biol. Sci..

[58] Tomás C., Vicient C.M. (2024). The genomic shock hypothesis: Genetic and epigenetic alterations of transposable elements after interspecific hybridization in plants. Epigenomes.

[59] Warschefsky E., Penmetsa R.V., Cook D.R., von Wettberg E.J.B. (2014). Back to the wilds: Tapping evolutionary adaptations for resilient crops through systematic hybridization with crop wild relatives. Am. J. Bot..

[60] Rajendrakumar P., Hariprasanna K., Seetharama N. (2015). Prediction of heterosis in crop plants-status and prospects. Am. J. Exp. Agric..

[61] Flavell R.B., O’Dell M., Hutchinson J. (1981). Nucleotide sequence organization in plant chromosomes and evidence for sequence translocation during evolution. Cold Spring Harb. Symp. Quant. Biol..

[62] Biscotti M.A., Olmo E., Heslop-Harrison J.S. (2015). Repetitive DNA in eukaryotic genomes. Chromosom Res..

[63] Meštrovi´c N., Mravinac B., Pavlek M., Vojvoda-Zeljko T., Šatovi´c E., Plohl M. (2015). Structural and functional liaisons between transposable elements and satellite DNAs. Chromosome Res..

[64] Badaeva E.D., Shelukhina O.Y., Dedkova O.S., Loskutov I.G., Pukhalsky V.A. (2011). Comparative cytogenetic analysis of hexaploid *Avena* L. species. Russ. J. Genet..

[65] Srivastava A.K., Schlessinger D. (1991). Structure and organization of ribosomal DNA. Biochimie.

[66] Garcia S., Kovařík A., Leitch A.R., Garnatje T. (2017). Cytogenetic features of rRNA genes across land plants: Analysis of the plant rDNA database. Plant J..

[67] Rogers S.O., Bendich A.J. (1987). Ribosomal RNA genes in plants: Variability in copy number and in intergenic spacer. Plant Mol. Biol..

[68] Linares C., González J., Ferrer E., Fominaya A. (1996). The use of double fluorescence *in situ* hybridization to physically map the positions of 5S rDNA genes in relation to the chromosomal location of 18S-5.8S-26S rDNA and a C genome specific DNA sequence in the genus *Avena*. Genome.

[69] Rodionov A.V., Amosova A.V., Krainova L.M., Machs E.M., Mikhailova Y.V., Gnutikov A.A., Muravenko O.V., Loskutov I.G. (2020). Phenomenon of multiple mutations in the 35S rRNA genes of the C subgenome of polyploid *Avena* L.. Rus. J. Gen..

[70] Winterfeld G., Döring E., Röser M. (2009). Chromosome evolution in wild oat grasses (Aveneae) revealed by molecular phylogeny. Genome.

[71] Tucker S., Vitins A., Pikaard C.S. (2010). Nucleolar dominance and ribosomal RNA gene silencing. Curr. Opin. Cell Biol..

[72] Craig S., Pikaard C.G., Chandrasekhara C., McKinlay A., Enganti R., Fultz D. (2023). Reaching for the off switch in nucleolar dominance. Plant J..

[73] Zhang X., Chen Y., Wang L., Yuan Y., Fang M., Shi L., Lu R., Comes H.P., Ma Y., Chen Y. (2023). Pangenome of water caltrop reveals structural variations and asymmetric subgenome divergence after allopolyploidization. Hortic. Res..

[74] Jellen E.N., Phillips R.L., Rines H.W. (1993). C-banded karyotypes and polymorphisms in hexaploid oat accessions (*Avena* spp.) using Wright’s stain. Genome.

[75] Jellen E.N., Gill B.S., Cox T.S. (1994). Genomic in situ hybridization differentiates between A/D- and C-genome chromatin and detects intergenomic translocations in polyploid oat species (genus *Avena*). Genome.

[76] Yang Q., Hanson L., Bennett M.D., Leitch I.J. (1999). Genome structure and evolution in the allohexaploid weed *Avena fatua* L. (Poaceae). Genome.

[77] Hayasaki M., Morikawa T., Tarumoto I. (2000). Intergenomic translocations of polyploid oats (genus *Avena*) revealed by genomic *in situ* hybridization. Genes Genet. Syst..

[78] Belyakov E.A., Machs E.M., Mikhailova Y.V., Rodionov A.V. (2019). The study of hybridization processes within genus *Sparganium* L. subgenus *Xanthosparganium* Holmb. Based on data of next generation sequencing (NGS). Ecol. Genet..

[79] Zhang M., Tang Y.W., Xu Y., Yonezawa T., Shao Y., Wang Y.G., Song Z.-P., Yang J., Zhang W.J. (2021). Concerted and birth-and-death evolution of 26S ribosomal DNA in *Camellia* L.. Ann. Bot..

[80] Gnutikov A.A., Nosov N.N., Loskutov I.G., Blinova E.V., Shneyer V.S., Rodionov A.V. (2023). Origin of wild polyploid *Avena* species inferred from polymorphism of the ITS1 rDNA in their genomes. Diversity.

[81] Wang X.-C., Liu C., Huang L., Bengtsson-Palme J., Chen H., Zhang J.-H., Cai D., Li J.-Q. (2015). ITS1: A DNA barcode better than ITS2 in eukaryotes?. Mol. Ecol. Res..

[82] Schultz J., Maisel S., Gerlach D., Müller T., Wolf M. (2005). A common core of secondary structure of 33 the internal transcribed spacer 2 (ITS2) throughout the Eukaryota. RNA.

[83] Coleman A.W. (2015). Nuclear rRNA transcript processing versus internal transcribed spacer 29 secondary structure. Trends Genet..

[84] Zhang X., Cao Y., Zhang W., Simmons M.P. (2020). Adenine· cytosine substitutions are an alternative 13 pathway of compensatory mutation in angiosperm ITS2. RNA.

[85] Brassac J., Blattne r.F.R. (2015). Species-level phylogeny and polyploid relationships in *Hordeum* (Poaceae) inferred by next-generation sequencing and in silico cloning of multiple nuclear loci. Syst. Biol..

[86] Rodionov A.V., Gnutikov A.A., Nosov N.N., Machs E.M., Mikhaylova Y.V., Shneyer V.S., Punina E.O. (2020). Intragenomic polymorphism of the ITS 1 region of 35S rRNA gene in the group of grasses with two-chromosome species: Different genome composition in closely related *Zingeria* species. Plants.

[87] Amosova A.V., Yurkevich O.Y., Rodionov A.V., Bolsheva N.L., Samatadze T.E., Zoshchuk S.A., Muravenko O.V. (2022). Repeatome Analyses and Satellite DNA Chromosome Patterns in *Deschampsia sukatschewii*, *D. cespitosa*, and *D. antarctica* (Poaceae). Genes.

[88] Gerlach W.L., Bedbrook J.R. (1979). Cloning and characterization of ribosomal RNA genes from wheat and barley. Nucleic Acids Res..

[89] Gerlach W.L., Dyer T.A. (1980). Sequence organization of the repeating units in the nucleus of wheat which contain 5S rRNA genes. Nucleic Acids Res..

[90] Ridgway K.P., Duck J.M., Young J.P.W. (2003). Identification of roots from grass swards using PCR-RFLP and FFLP of the plastid trnL (UAA) intron. BMC Ecol..

[91] White T.J., Bruns T., Lee S., Taylor J., Innis M.A., Gelfand D.H., Sninsky J.J., White T.J. (1990). Amplification and direct sequencing of fungal ribosomal RNA genes for phylogenetics. PCR Protocols: A Guide to Methods and Applications.

[92] Bolger A.M., Lohse M., Usadel B. (2014). Trimmomatic: A flexible trimmer for Illumina sequence data. Bioinformatics.

[93] Okonechnikov K., Golosova O., Fursov M., the UGENE team (2012). Unipro UGENE: A unified bioinformatics toolkit. Bioinformatics.

[94] Rognes T., Flouri T., Nichols B., Quince C., Mahe F. (2016). VSEARCH: A versatile open source tool for metagenomics. PeerJ.

[95] Clement M., Posada D., Crandall K.A. (2000). TCS: A computer program to estimate gene genealogies. Mol. Ecol..

[96] Múrias dos Santos A., Cabezas M.P., Tavares A.I., Xavier R., Branco M. (2016). tcsBU: A tool to extend TCS network layout and visualization. Bioinformatics.

[97] Ronquist F., Teslenko M., van der Mark P., Ayres D.L., Darling A., Höhna S., Larget B., Liu L., Suchard M.A., Huelsenbeck J.P. (2012). MrBayes 3.2: Efficient Bayesian phylogenetic inference and model choice across a large model space. Syst. Biol..

[98] Tamura K., Stecher G., Kumar S. (2021). MEGA11: Molecular Evolutionary Genetics Analysis version 11. Mol. Biol. Evol..

