# Supplementary material for: Genome Variability in Artificial Allopolyploid Hybrids of Avena sativa L. and Avena macrostachya Balansa ex Coss. et Durieu Based on Marker Sequences of Satellite DNA and the ITS1–5.8S rDNA Region
Source: Int J Mol Sci. 2024 May 19;25(10):5534. doi: 10.3390/ijms25105534 (PMC11122565; doi:10.3390/ijms25105534)
Supplement: Supplementary file 1 [file ijms-25-05534-s001.zip › ijms-2997392 Supplementary Figures.pdf]

# Supplementary Figures

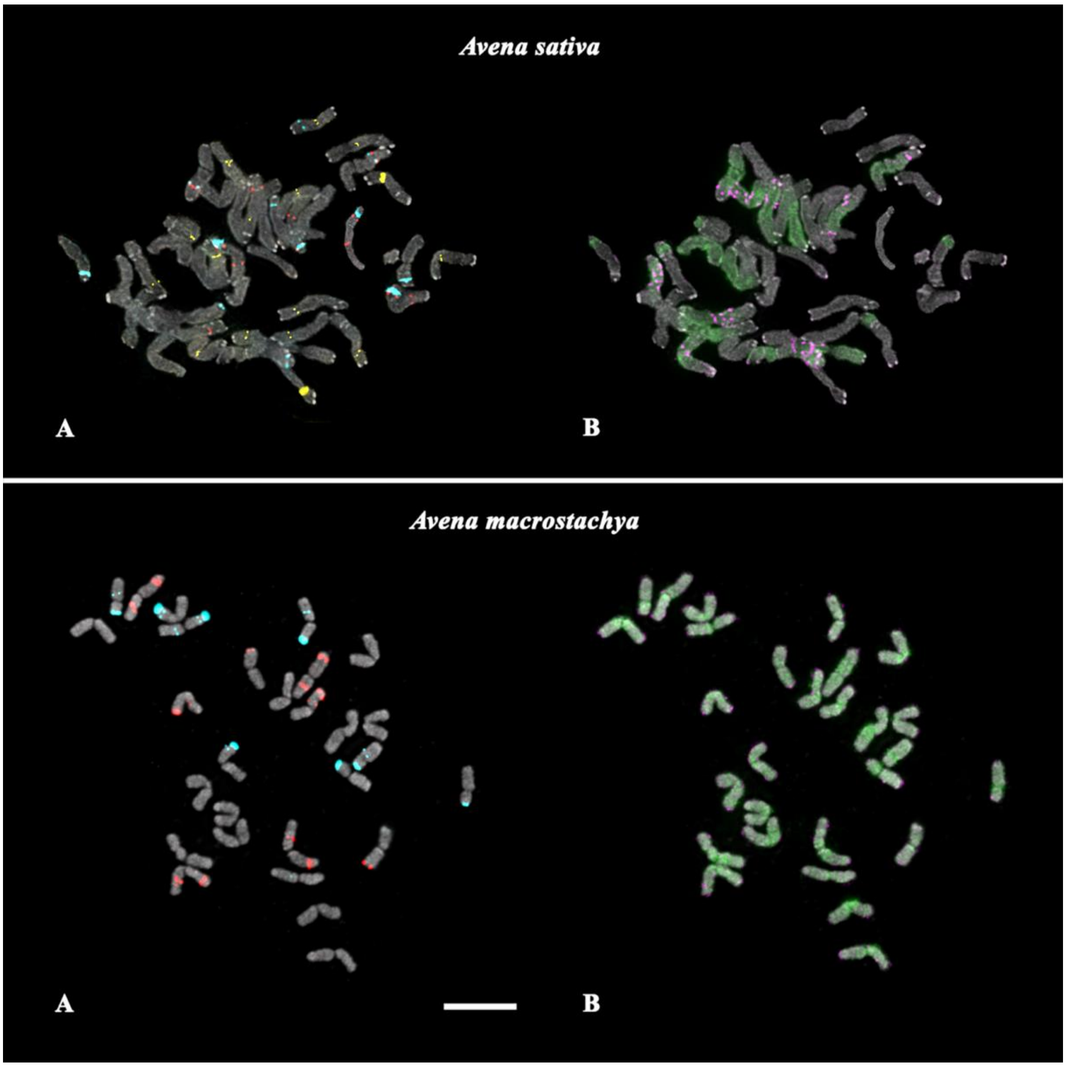


**Figure S1.** FISH-based localization of (A) 35S rDNA (aqua), 5S rDNA (red) and GTT (yellow) and also (B) 6C343 (purple) and 6C51 (green) signals on chromosomes of *Avena sativa* and *Avena macrostachya.* DAPI-staining – grey. Scale bar – 5 μm.

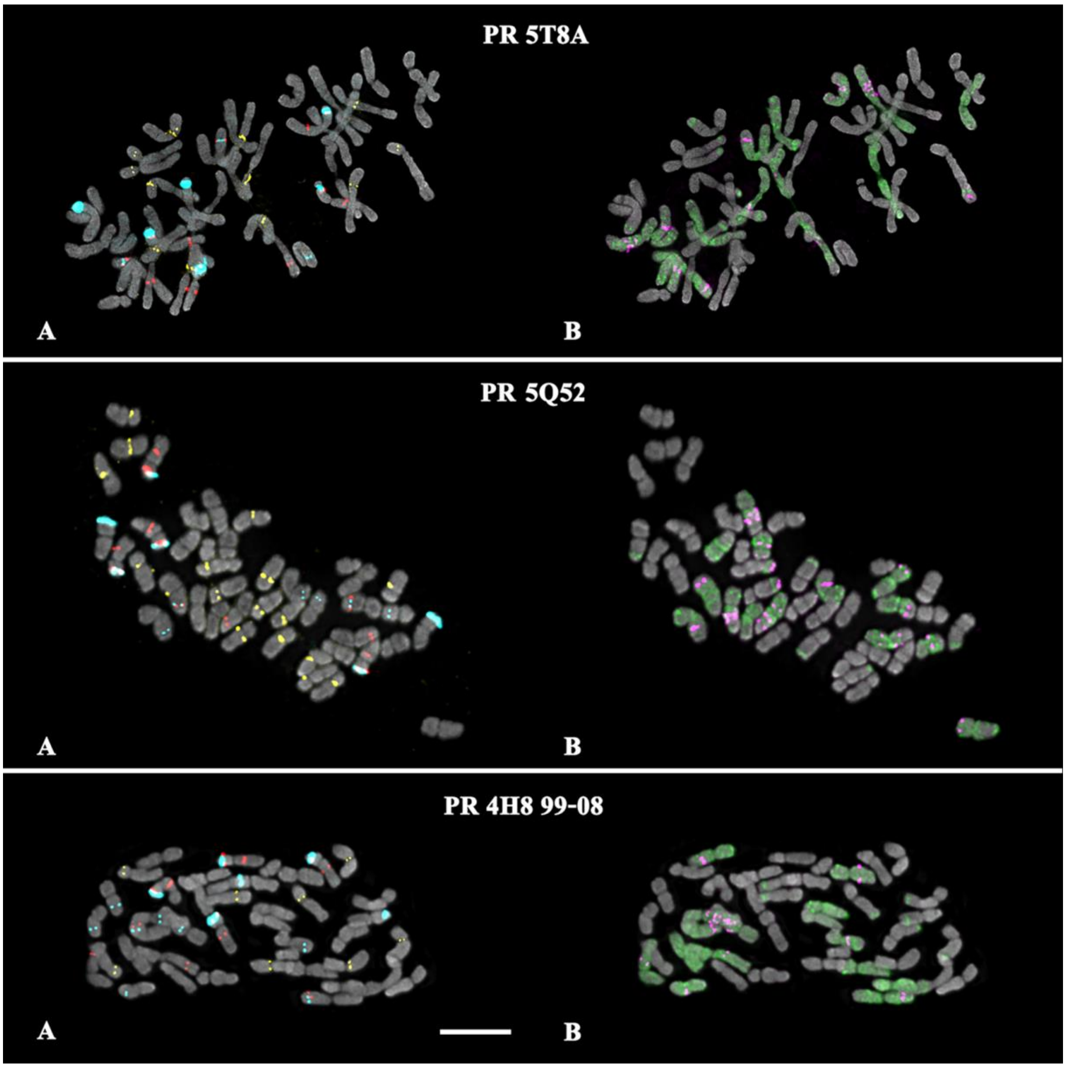


**Figure S2.** FISH-based localization of (A) 35S rDNA (aqua), 5S rDNA (red) and GTT (yellow) signals and also (B) 6C343 (purple) and 6C51 (green) signal on chromosomes of the studied hexaploid hybrids. DAPI-staining – grey. Scale bar – 5 μm.

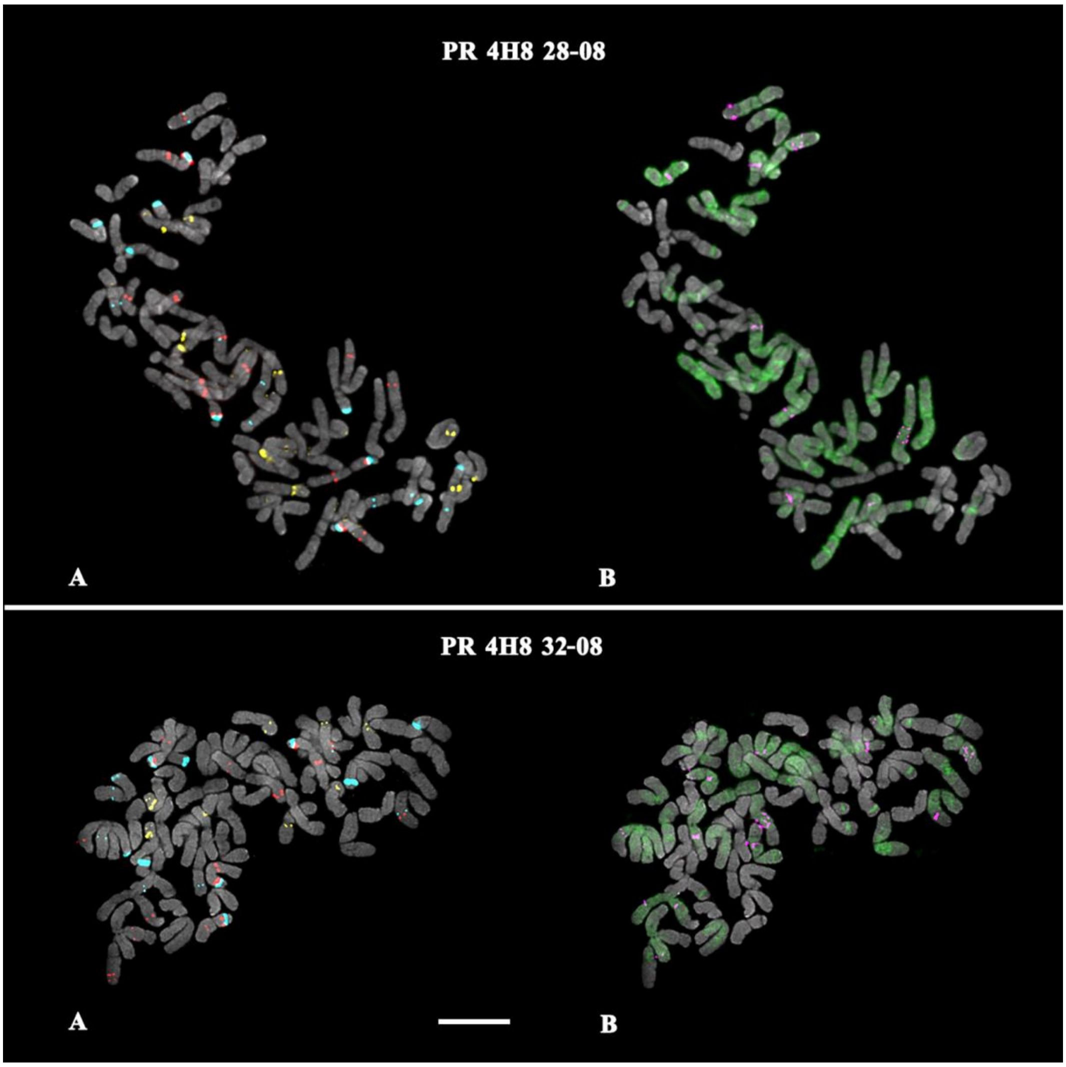


**Figure S3.** FISH-based localization of (A) 35S rDNA (aqua), 5S rDNA (red) and GTT (yellow) signals and also (B) 6C343 (purple) and 6C51 (green) signal on chromosomes of the studied octoploid hybrids PR 4H8 28-08 and PR 4H8 32-08. DAPI-staining – grey. Scale bar – 5 µm.

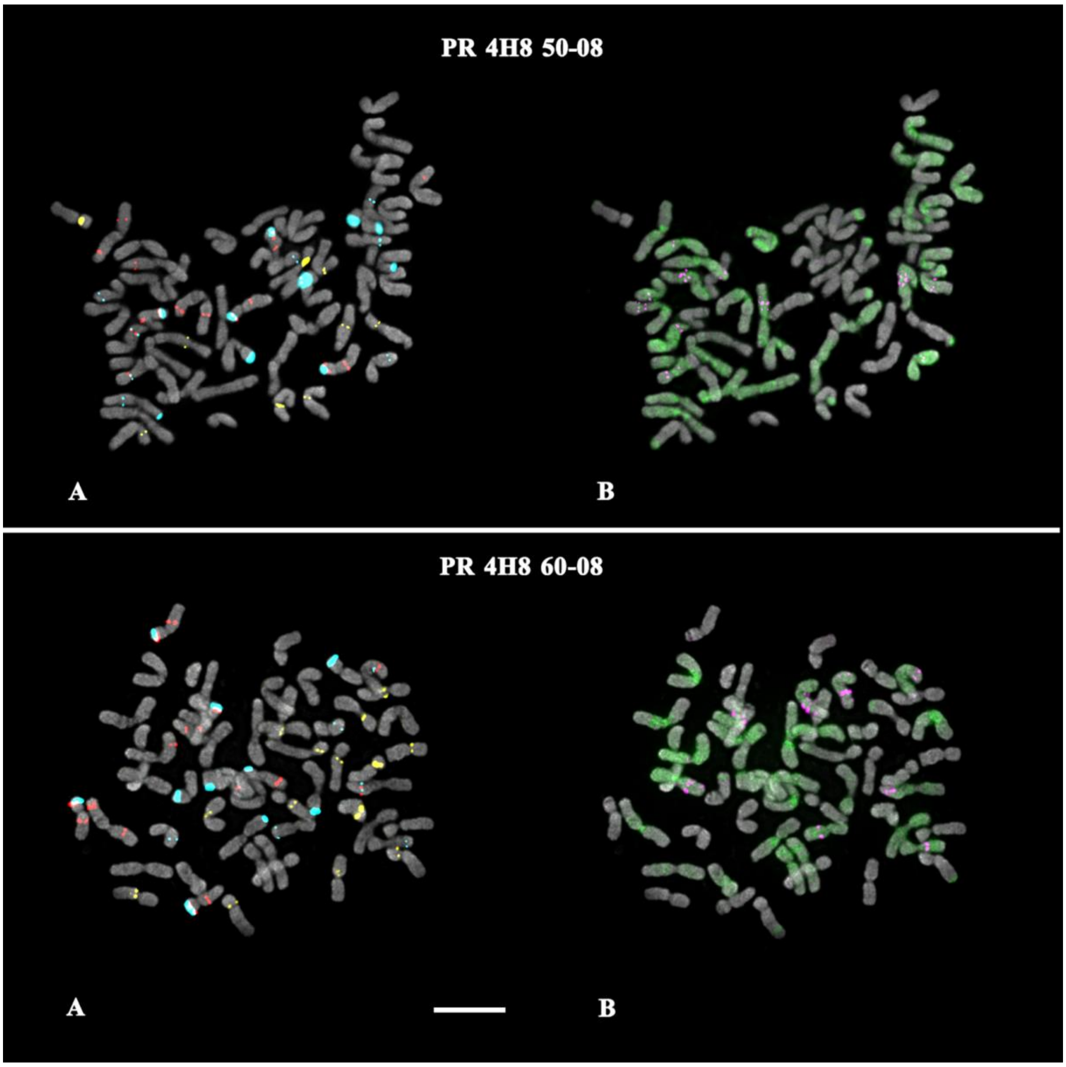


**Figure S4.** FISH-based localization of (A) 35S rDNA (aqua), 5S rDNA (red) and GTT (yellow) signals and also (B) 6C343 (purple) and 6C51 (green) signal on chromosomes of the studied octoploid hybrids PR 4H8 50-08 and PR 4H8 60-08. DAPI-staining – grey. Scale bar – 5 μm.

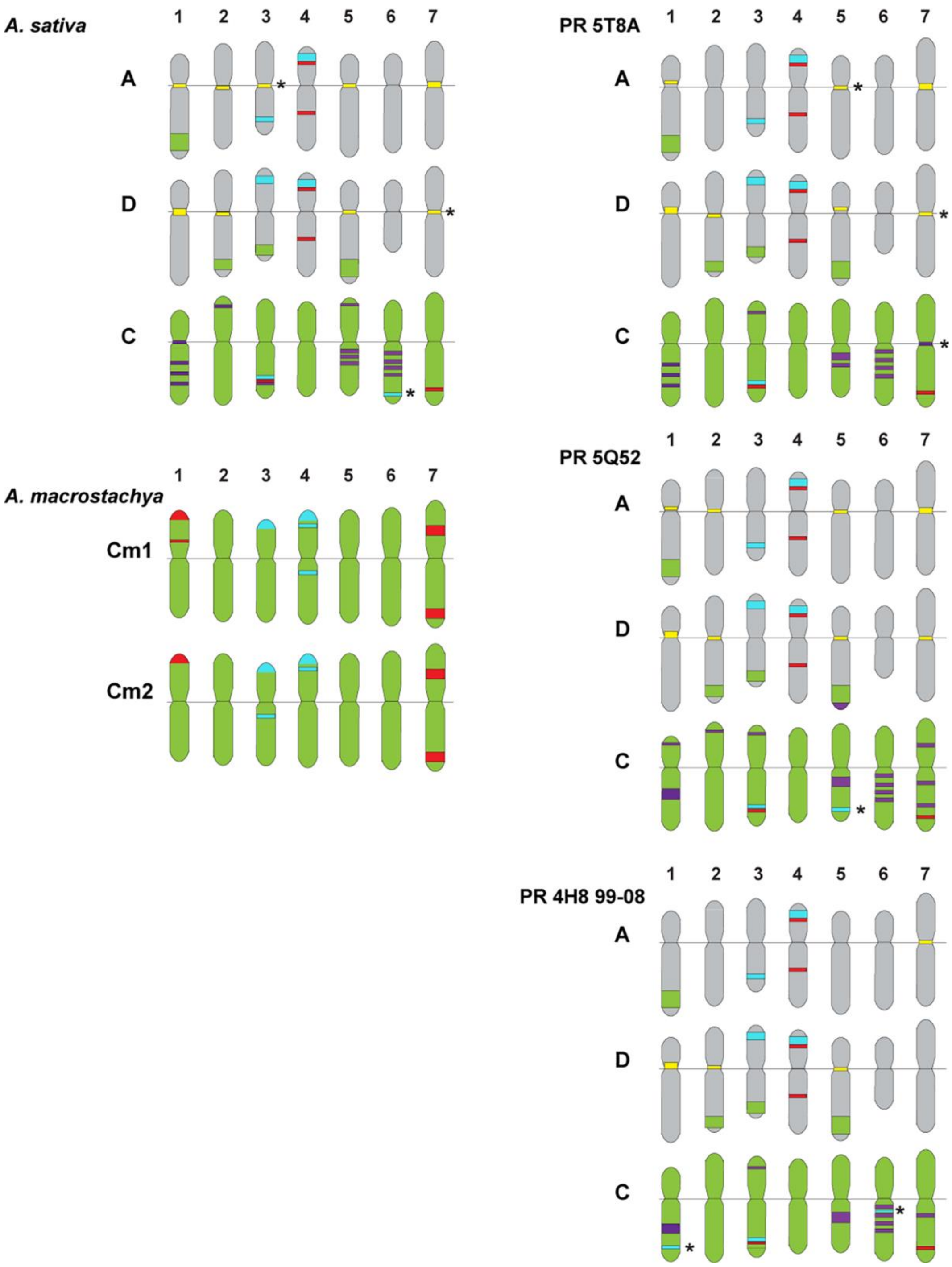


**Figure S5.** Patterns of distribution of 35S rDNA (aqua), 5S rDNA (red), GTT (yellow), 6C343 (purple) and 6C51 (green) signals on chromosomes of *Avena sativa*, *Avena macrostachya* and their hexaploidy hybrids PR 5T8A, 5Q52, and PR 4H8 99-08. Asterisks denote polymorphic DNA loci.

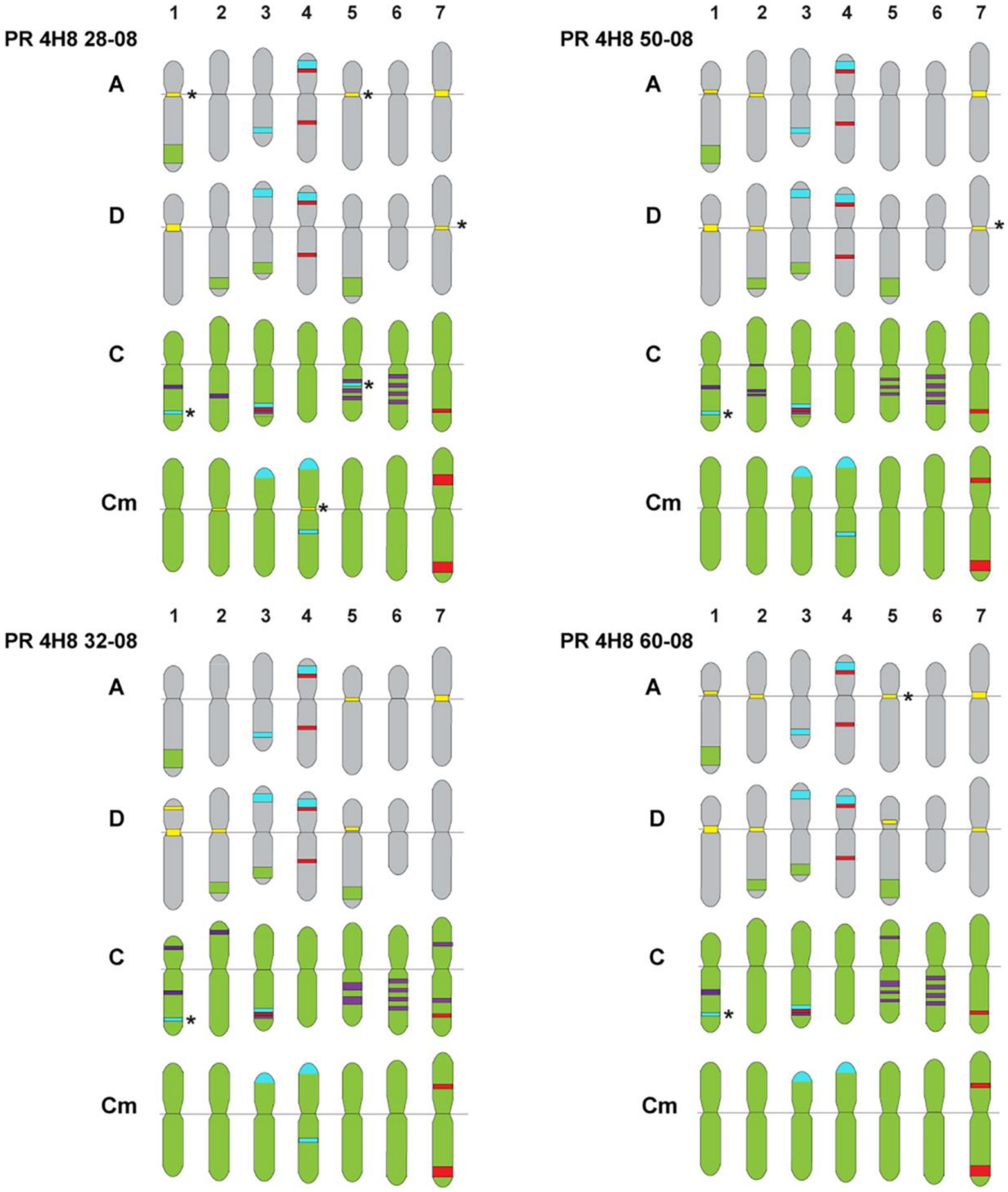


**Figure S6.** Patterns of distribution of 35S rDNA (aqua), 5S rDNA (red), GTT (yellow), 6C343 (purple) and 6C51 (green) signals on chromosomes of the studied octoploid hybrids PR 4H8 28-08, PR 4H8 32-08, PR 4H8 50-08 and PR 4H8 60-08. Asterisks denote polymorphic DNA loci.
